# Evaluation of ozonated and ultrasonically treated corn starch as an adsorbent for patulin in buffer solutions

**DOI:** 10.1038/s41598-025-85108-w

**Published:** 2025-01-17

**Authors:** Tarek A. El-desouky

**Affiliations:** Department of Food Toxicology and Contaminant, National Research Centre, Dokki, Giza, Egypt

**Keywords:** Patulin, Adsorbent, Corn starch, Ozonation, Ultrasonic, Polymer chemistry, Biochemistry, Carbohydrates

## Abstract

This study evaluates the potential of ozonated corn starch (OCS) and ultrasonicated ozonated corn starch (USOCS) as adsorbents for patulin removal in buffer solutions. The results indicated that dual modification significantly altered the starch’s structure, introducing functional groups such as carbonyl and carboxyl groups, and increasing its surface area. These modifications led to enhanced patulin adsorption capacity. Adsorption efficiency was tested across different adsorbent doses (150 mg, 200 mg, 250 mg) and contact times (15, 30, 45, and 60 min). The highest removal efficiency of 92.5% was recorded for the 250 mg dose at 60 min, with USOCS showing superior performance compared to native corn starch and OCS. Kinetic studies revealed that the pseudo-second-order model provided the best fit for the adsorption process, indicating chemisorption as the dominant mechanism. The Langmuir and Freundlich isotherms were used to describe the adsorption behavior, with a maximum adsorption capacity (*q*_*max*_) of 15.19 µg/mg and a Langmuir constant (*K*_*L*_) of 54.00 L/µg for the 250 mg dose. Additionally, the modified starch demonstrated consistent adsorption performance at varying concentrations, with a favorable adsorption intensity (*n* > 1), supporting its potential for practical applications. These findings highlight the modified corn starch as an efficient, biodegradable, and low-cost adsorbent suitable for mitigating patulin contamination in food products, offering a sustainable alternative for improving food safety.

## Introduction

Patulin (PAT), a mycotoxin produced by over 60 fungal species from more than 30 genera^1,2^, is commonly associated with fungi like *Penicillium expansum*, as well as species from the *Aspergillus* and *Byssochlamys* genera. Due to its water solubility and heat stability, PAT can easily infiltrate and persist in processed food products^3^. Exposure to PAT causes serious health risks to both humans and animals, including mutagenic, teratogenic, and carcinogenic effects^4^. PAT contamination in fruit products has been reported globally, with studies showing varying detection rates and concentrations across different regions. Funes and Resnik (2009) detected PAT in 22.2% of apple products, with an average concentration of 123 µg/kg, and in 16.7% of pear products^5^. Papayas from Portugal also exhibited a 50% detection rate, with the highest level reaching 118.3 µg/kg^6^. In Tunisia, PAT levels in apple juice ranged from zero to 167 µg/L, while a separate study reported contamination as high as 889 µg/L^7,8^. In China, Ji et al. (2017) analyzed 137 fruit products, including dried fruits, juices, and jams, finding that 30.7% of samples were contaminated with PAT, with concentrations ranging from 10.0 to 276.9 µg/kg. Dried figs and dried longans exhibited the highest contamination levels, while fruit juices and jams had lower average concentrations. Notably, 17.5% of the samples exceeded the EU’s maximum permissible limit for PAT^9^. In Serbia, an investigation into PAT occurrence in fruit juices assessed risks for children, adolescents, and adults. It was found that 51.4% of fruit juice samples contained PAT, with apple juices showing significantly higher contamination levels (74.0%) compared to multi-fruit juices (27.5%). The average PAT concentration in apple juices was 6.4 µg/kg, much higher than the 2.1 µg/kg in multi-fruit^10^. In Belgium, testing of 103 juices, 42 purees, and 10 ciders revealed PAT in 54.4% of apple juices (up to 191.1 µg/L) and 7.1% of purees (up to 35.9 µg/kg). No PAT was detected in cider samples, but five apple juices and one puree exceeded the maximum levels set by EU Regulation EC 1881/2006 for infants and young children^11^. A separate study tested 442 fruit samples, including oranges, apples, apricots, lemons, and guava, for PAT contamination using HPLC. It was found that 17%, 23%, and 28% of samples were positive for PAT at the farm, transportation, and market stages, respectively. Notably, 56% of transportation samples and 41% of market samples exceeded the 50 μg/kg threshold^12^. These studies indicate widespread PAT contamination across various fruit products and regions, highlighting the need for continuous monitoring and risk assessment to ensure food safety. Furthermore, they emphasize the importance of addressing the combined effects of multiple toxic substances, as PAT contamination in food may not be a significant risk alone, but could pose a greater threat when present alongside other contaminants. As a result, many countries worldwide have established a maximum permissible level of 50 µg/kg in food. Since 1995, the Joint FAO/WHO Expert Committee on Food Additives has recommended a provisional maximum tolerable daily intake of 0.4 µg/kg body weight per day^13,14^. PAT contamination of agricultural products significantly affects product quality and causes economic losses for producers and exporters. While common food processing techniques like pasteurization, filtration, and fermentation can reduce PAT levels, they are not sufficient on their own to ensure complete removal. Many existing mitigation methods are limited by high costs, specialized equipment, energy or reagent demands, and the need for trained personnel^15,16^. As a result, we need to develop strategies to reduce PAT. Adsorption has been identified as one of the most efficient methods for removing contaminants^17^, and researchers have increasingly focused on developing safe, effective, and reliable adsorption materials to reduce PAT levels in recent years. However, many of these materials are costly, require advanced equipment, or involve complex operational procedures. Corn starch, being a natural, biodegradable, and abundant material, offers a more sustainable and eco-friendly alternative. Through chemical and physical modifications, the functional properties of corn starch can be significantly enhanced, making it a more effective adsorbent for PAT removal^18,19^. Starch can be modified by physical, chemical, enzymatic, and combined ways to improve its properties^20,21^. Ozonation and ultrasonic treatments are two promising methods for modifying starch. In 1997, the US Food and Drug Administration (FDA) classified ozone gas as a GRAS substance. Then, in 2001, it was authorized for use as a food sanitizer that can come into direct contact with food^22^. During oxidative modification, the hydroxyl groups (OH) on starch are initially converted into carbonyl groups (C=O), which are then further oxidized into carboxyl groups^23^. Oxidation primarily affects the hydroxyl groups located at the C-2, C-3, and C-6 positions of the starch^24^. Ultrasonics has an effect on starch through increasing the surface area and generating porous structures. In this study, ozonated cornstarch (OCS) was prepared from native corn starch (NCS), followed by ultrasonic treatment to produce ultrasonicated ozonated corn starch (USOCS). The structural and functional changes resulting from dual modification were investigated. The removal efficiencies (RE) of NCS, OCS, and USOCS were compared by evaluating patulin (PAT) removal at varying adsorbent doses (150 mg, 200 mg, and 250 mg) and contact times (15, 30, 45, and 60 min). The adsorption kinetics of PAT on USOCS were assessed using pseudo-first-order and pseudo-second-order models to identify the dominant adsorption mechanism. Additionally, the adsorption behavior of PAT on USOCS was analyzed using Langmuir and Freundlich isotherms, with key parameters such as the maximum adsorption capacity (*q*_*max*_) and the Langmuir constant (*K*_*L*_) calculated.

## Materials and methods

### Chemicals

Patulin (analytical standard, purity ≥ 98%) was purchased from Sigma Chemical Co. (St. Louis, MO, USA). The ultrapure water used in this study was produced using the Millipore-Q SP Reagent Water system (Millipore, Bedford, MA, USA). Chromatography-grade ethyl acetate and acetonitrile were purchased from Aldrich Chemical Co., Milwaukee, WI. Other chemicals were of analytical grade.

### Preparation of ozonation Corn Starch (OCS)

OCS was prepared from NCS using an ozone generator (Model OZO 6 VTTL) by mixing NCS, obtained from the Egyptian Starch and Glucose Company (ESGC), with water at a 1:9 ratio and treating it with ozone gas at a rate of 30 g/h for 1 h. After centrifugation, the resulting OCS was air-dried at 40 °C for 2 h and then subjected to ultrasonication to prepare USOCS.

### Preparation of USOCS

A 100 mL suspension of OCS was prepared at a concentration of 10% (w/w) in water. The suspension was then subjected to ultrasonication for 20 min at 40 kHz using a fixed probe sonicator (Q55-Qsonica, USA). To ensure consistent processing conditions, the temperature was maintained at 25°C throughout the ultrasonication process by using a water bath.

### Characterization

Various techniques, such as Fourier Transform Infrared (FTIR) spectroscopy, Thermogravimetric Analysis (TGA), Scanning Electron Microscopy (SEM), and Energy-Dispersive X-ray Spectroscopy (EDX), are employed to characterize NCS, OCS, and USOCS, along with the determination of carbonyl and carboxyl content in OCS and USOCS^25^.

### Assay adsorption of patulin in contaminated PBS

To each 100 mL of phosphate-buffered saline (PBS) contaminated artificially to contain 100 ug of PAT standard, the NCS, OCS, and OSNPs will be added individually at different weights (150, 200, and 250 mg/100 ml), then the samples will be left for periods ranging from 15, 30, 45, and 60 min at 25 °C. Finally, PAT toxin was extracted according to the Funes et al. (2013) method. In this method, 10 ml of ethyl acetate was added to 10 ml of PBs samples, which were extracted three times with 10 ml of ethyl acetate. Extracts were gathered and evaporated to dryness under N2^26^.

#### HPLC conditions for PAT

The residue was dissolved in 300 μl acetic acid (0.1% v/v) and was injected (50 μl) in the HPLC Waters 2690 XE Separations Module system, 2487 UV detector, and the data were integrated and recorded by Millennium Chromatography Manager software 210 (Waters, Milford MA 01757). The mobile phase acetonitrile: water (10: 90 v/v), flow rate 0.8 ml/min, reverse phase hyperclone 5μ ODS C_18_ column (2.5 mm × 30 cm), and UV detector with wavelength 276 nm^27^. The removal efficiency (RE) of PAT was calculated by the equation below:

$${\text{RE}} = \frac{Co - Ce}{{Co}} \times 100$$

where Co and C_e_, are the initial and final concentration of PAT (µg/L), respectively.

### Method validation

Specificity, linearity (R^2^), limit of detection (LOD), limit of quantification (LOQ), and recovery rates were assessed as part of the method performance and validation process, in compliance with Commission Regulation (EC) No 401/2006 (European Commission, 2006). The extraction and analysis steps were carried out as outlined earlier^28^

#### Accuracy

The method’s accuracy was assessed through recovery studies, where samples were spiked with PAT at six concentration levels, analyzed in triplicate, and average percentage recoveries calculated.

$${\text{Recovery}}\,\% = \frac{{{\text{Actual\,amount\,of\,PAT}} }}{{{\text{Initial\,amount\,of\,PAT \,was\,added}}}} \times 100$$

#### Linearity

In method validation, linearity is essential because it offers a clear process for figuring out the quantities of active constituents in a composite system. The formula for calculating it is y = mx + b. where (b) is the line’s y-intercept, or the point at which it crosses the y-axis, and (m) is the line’s gradient or slope.

#### LOD and LOQ

In this study, the LOD is defined as the minimum amount of PAT in a sample that can be detected but not quantitated accurately. While LOQ is the minimum amount of PAT in a sample that can be accurately and precisely quantified.

### Statistical analysis

All of the validation and adsorption experiments were performed in triplicate; the results were presented as means ± standard deviation.

## Results and discussion

### FTIR analysis

**Fig. 1:**
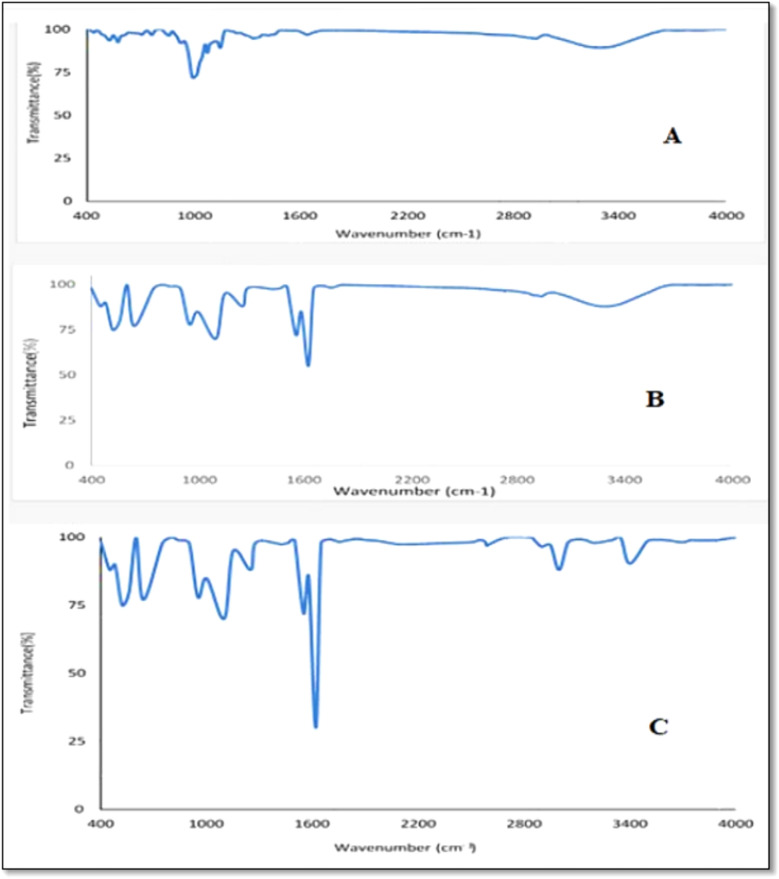
The FTIR spectroscopy of the NCS (**A**), OCS (**B**), and USOCS (**C**).

In this study, we used Fourier transform infrared spectroscopy (FTIR 6100; Perkin-Elmer), and the spectra were scanned in the 400–4000 cm^-1^ range at a resolution of 4 cm for analyzing chemical and structural changes that occur in NCS components due to ozonation and ultrasonic treatments, as represented in (Fig. 1). Ozonation of NCS leads to new oxygen-containing functional groups like carbonyl and carboxyl because of substitution of hydroxyl groups present in the starch polymers. As the ozonation process continues, the starch molecules undergo depolymerization through the cleavage of glycosidic bonds^29,30^**.** The broad bands observed around 3400–3600 cm⁻^1^ are due to the O–H stretching vibration of hydroxyl groups in the glucose units of starch, which appear because of ultrasonic treatment. This treatment leads to the breaking of the starch polymer into glucose units, as shown in Fig. 1C. In native starch, the O–H stretching band typically appears as a broad peak in this region (Fig. 1A). After ultrasonic treatment, this peak may become narrower or show a shift in intensity due to changes in the hydrogen-bonding network. Ultrasound partially disrupts the crystalline structure, weakening intermolecular hydrogen bonds, which alters the hydroxyl stretching behavior^31,32^. Additionally, the C=O carbonyl group exhibits absorption in the region of 1680–1750 cm⁻^1^^33^, while C–H stretching is observed in the 2800–3000 cm⁻^1^ region. These bands may shift slightly or decrease in intensity depending on the degree of ozonation and the conversion of hydroxyl groups into carbonyl or carboxyl groups, as shown in Fig. 1B. On the other hand, some studies suggested that the peak at 900 to 1080 cm^−1^ indirectly demonstrated the α-1,6 linkage and the α-1,4 linkage, which change after treating NCS with ozone gas and ultrasonic as in OCS and USOCS, respectively^25,34,35^.

### Impact of ozonation and ultrasonic on carbonyl and carboxyl group content

**Fig. 2:**
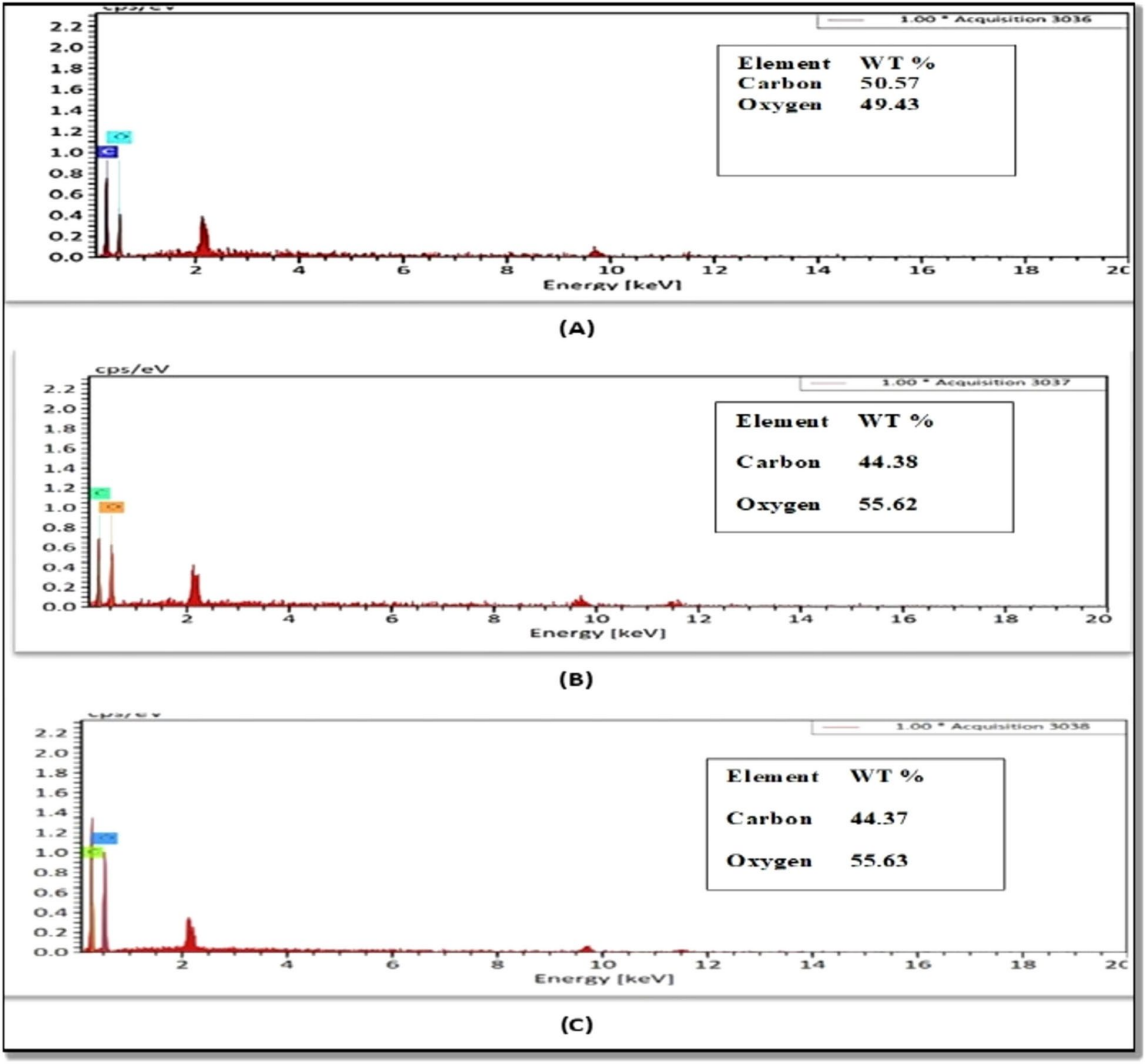
The EDX analysis of the NCS (**A**), OCS (**B**), and USOCS (**C**).

Table 1 presents the carbonyl, carboxyl contents of USOCS, and OCS, indicating that oxidation with ozone gas typically enhances these groups. The starch oxidation process involves two key reactions: the transformation of hydroxyl (-OH) groups into carboxyl (-COOH) and carbonyl (C=O) groups, as well as the depolymerization of starch molecules through glycosidic bond cleavage^29,36^. The findings show that the carboxyl content in OCS and USOCS is greater than the carbonyl content, likely because –OH groups are oxidized first^33^. Additionally, glucose chains in ozone-oxidized starches are preferentially oxidized at the C-6 position, leading to an increase in carbonyl groups^37,38^. Furthermore, SEM/EDX analysis (Fig. 2) corroborates these results by revealing changes in NCS.

**Table 1:** Carbonyl and carboxyl contents of OCS and USOCS.

Sample	Carbonyl content (CO/100 GU)	Carboxyl content (COOH/100 GU)
OCS	0.163	0.875
USOCS	0.135	0.736

### Thermogravimetric analysis (TGA)

**Fig. 3:**
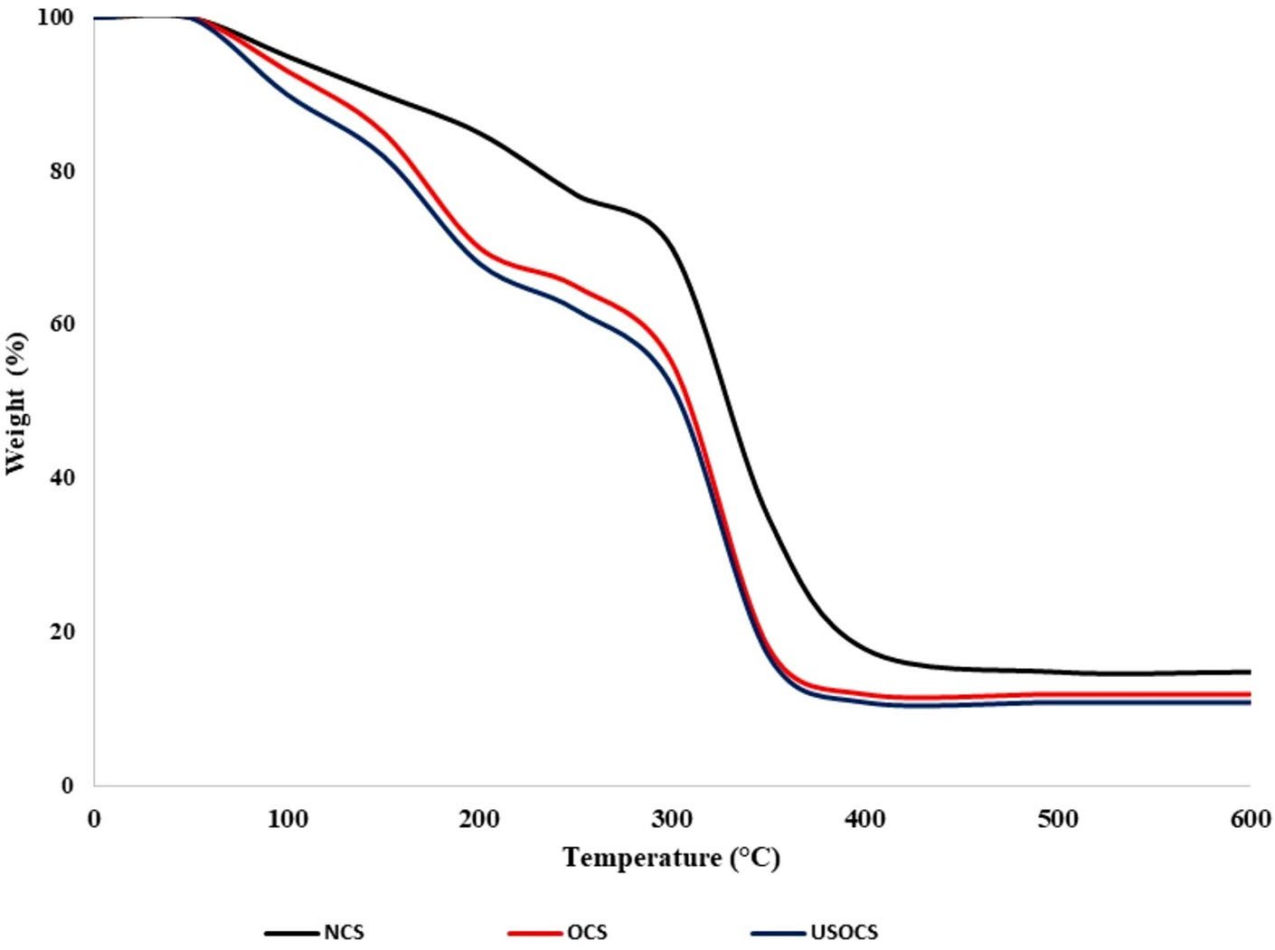
Thermogravimetric curves (TGA) for NCS, OCS and USOCS.

The thermal stability of NCS, OCS, and USOCS has been studied by thermogravimetric analysis (TGA, Shimadzu DTG-60, Japan) using an STA 6000 Perkin Elmer Analyzer from 50 °C to 600 °C at a heating rate of 10 °C/min under argon^39^. Figure 3 displays the TGA curves. A three-step weight loss was observed in all the samples. The first decomposition, between 50 and 100 °C, was due to the evaporation of free water remaining in the samples. The second stage (100–200 °C) was due to bound water and glycerol evaporation. The third was observed at 350 °C. After the final thermal destruction, the residual percentages at 600 °C of the NCS, OCS, and USOCS were 12, 21, and 17%, respectively. In general, OCS and USOCS begin to degrade at slightly lower temperatures compared to NCS. Numerous studies have demonstrated that oxidation degrades amylose and amylopectin, which results in reduced thermal stability^40–42^. The depolymerization of starch nanocrystals starts earlier than in NCS, and the increased specific surface area of starch nanocrystals significantly affects their thermal behavior^43^. Zhang et al. (2009) reported that the initial thermal decomposition temperature decreased from 276 °C for native starch to 221 °C for the oxidized form. The study indicates that both OCS and USOCS experience thermal degradation at lower temperatures, with OCS being more prone to thermal breakdown due to oxidative modifications^44^. While the thermal degradation of USOCS is attributed to their reduced molecular size and increased surface area, which make the modified starch more thermally sensitive initially. Additionally, the residual mass after thermal decomposition tends to be higher in OCS and USOCS because of the formation of more thermally stable oxidized by-products and cross-linking, leading to more char formation during decomposition^30,45^.

### Scanning electron microscopy (SEM) for NCS modified with ozonation and ultrasonic

**Fig. 4:**
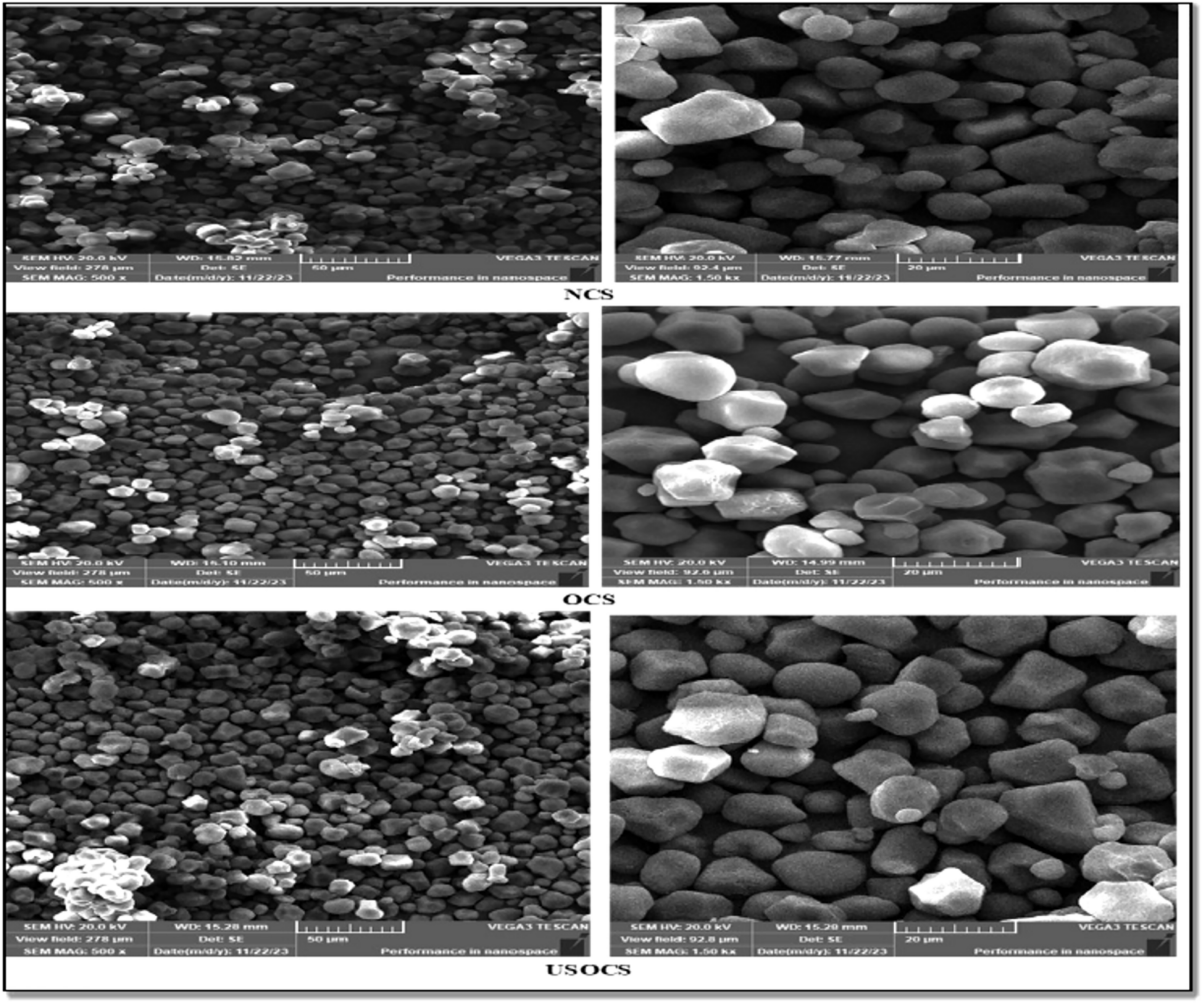
SEM micrographs of NCS, OCS, and USOCS.

To investigate the surface characteristics of OCS and USOCS, SEM was used with Model Tescan Vega 3 SBU attached to an EDX Unit at an acceleration voltage of 20 kV. As shown in Fig. 4, the SEM micrographs revealed that the NCS granules structure were smooth and round or oval shaped. In contrast, OCS granules exhibited rough, fibrous surfaces due to ozonation. For USOCS, the granules showed broken surfaces, and fissures, resulting from the ultrasonication process, which led to more irregular and rough surface textures. The modifications can break down the starch granules, altering their structure and making them more porous, which can be beneficial for certain functional properties, such as increased reactivity and better adsorption capacity. Which is consistent with the results of other authors^46–49^.

### Method Performance

**Fig. 5:**
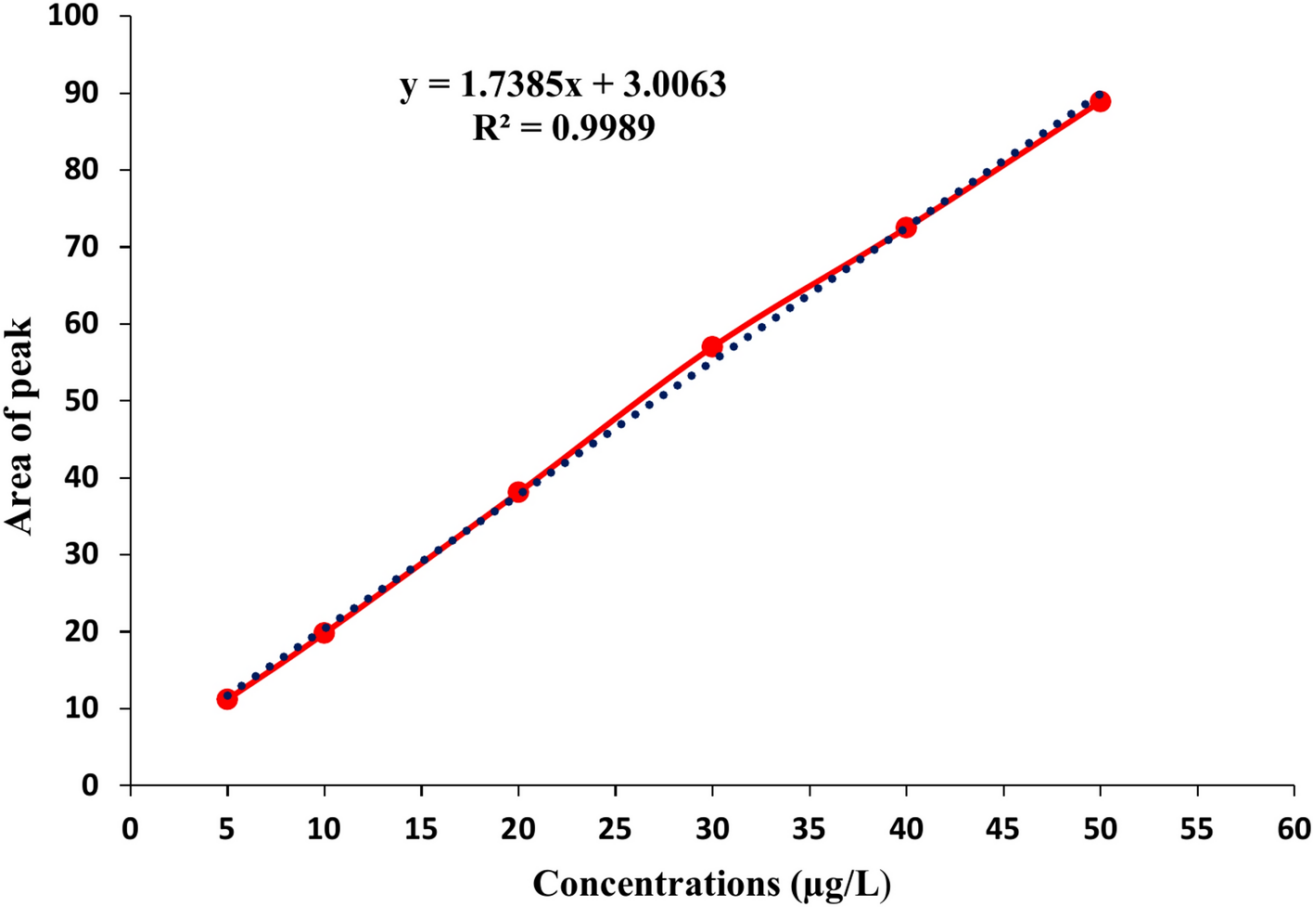
The calibration curve for the PAT by HPLC for evaluation of the linearity of the method.

The calibration curve shown in Fig. 5 for PAT was linear, with an R^2^ of 0.9989. On the other hand, recoveries studies were determined at six contamination levels (5, 10, 20, 30, and 50 and 140 µg/L), each level by triplicate. Data displayed in Table 2 the recovery percentages range from 72.7% at 5 µg/L to 94.06% at 50 µg/L. The lowest recovery (72.7%) at the lowest concentration may indicate potential losses or limitations in sensitivity at low levels, which is common in analytical methods. SD values are all below 3%, which demonstrates excellent precision. Regarding the LOD and LOQ values, they were recorded at 1.26 µg/L and 3.61 µg/L, respectively.

**Table 2:** Recoveries of patulin (PAT) from Contaminated PBs.

PAT (µg/L)	Recovery %	SD	RSD
5	72.7	2.04	2.81
10	80.3	1.52	1.89
20	89.9	1.25	1.39
30	91.6	1.75	1.91
40	94.03	1.71	1.82
50	94.06	1.3	1.38

*SD* standard deviation, *RSD* relative standard deviation % (SD/mean of recovery) × 100.

### In vitro study on the assessment of PAT removal

#### Effect of contact time and adsorbent dose on the efficiency of PAT removal

**Fig. 6:**
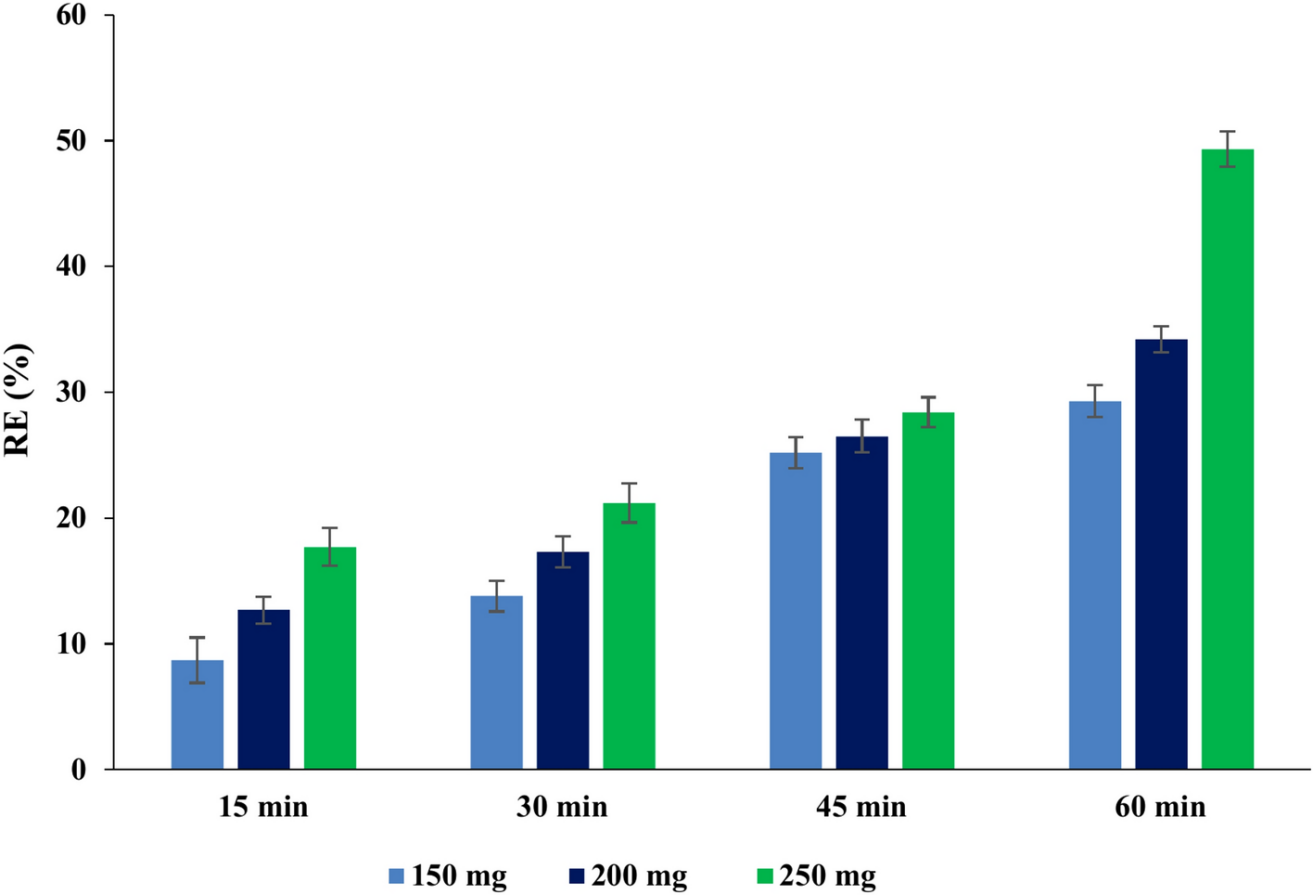
Impact of contact time and adsorbent dose on PAT removal efficiency by NCS.

**Fig. 7:**
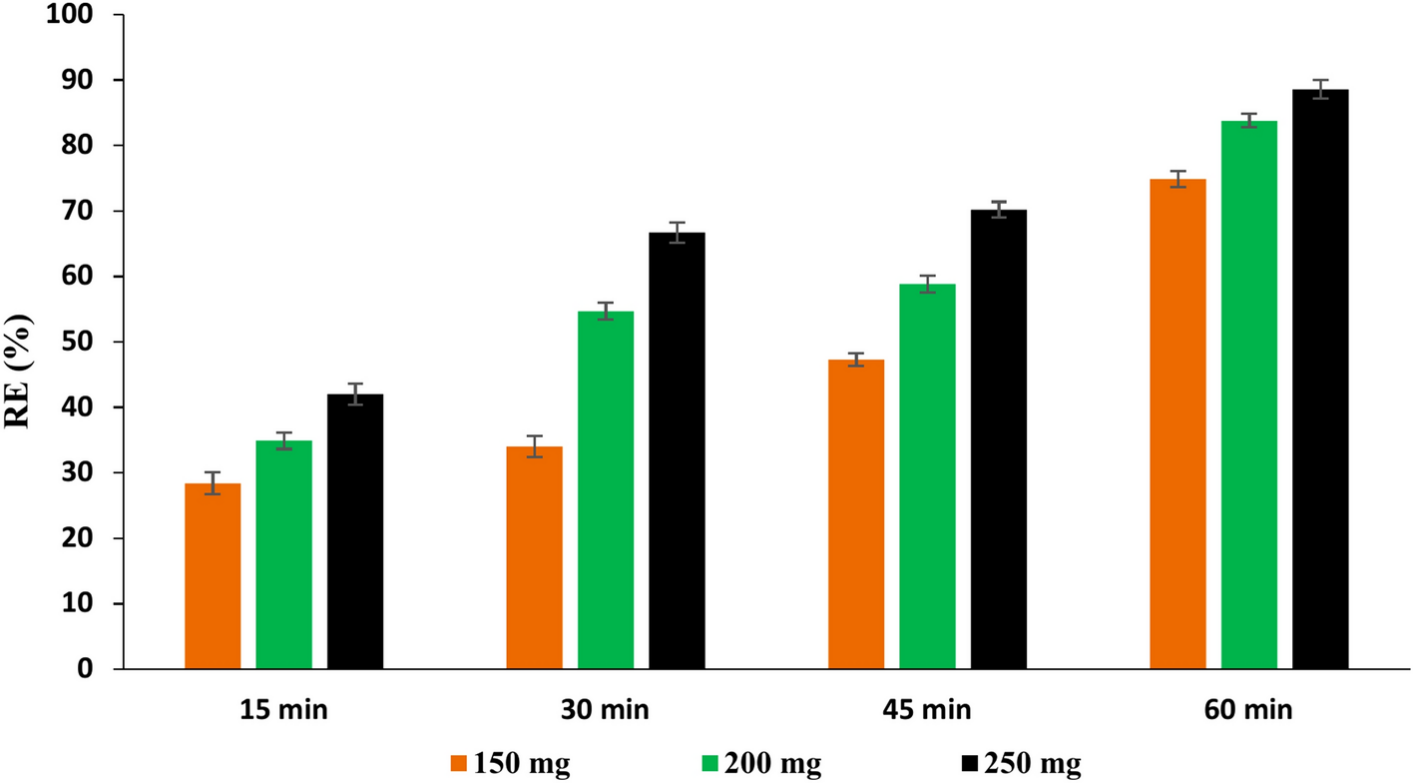
Impact of contact time and adsorbent dose on PAT removal efficiency by OCS.

**Fig. 8:**
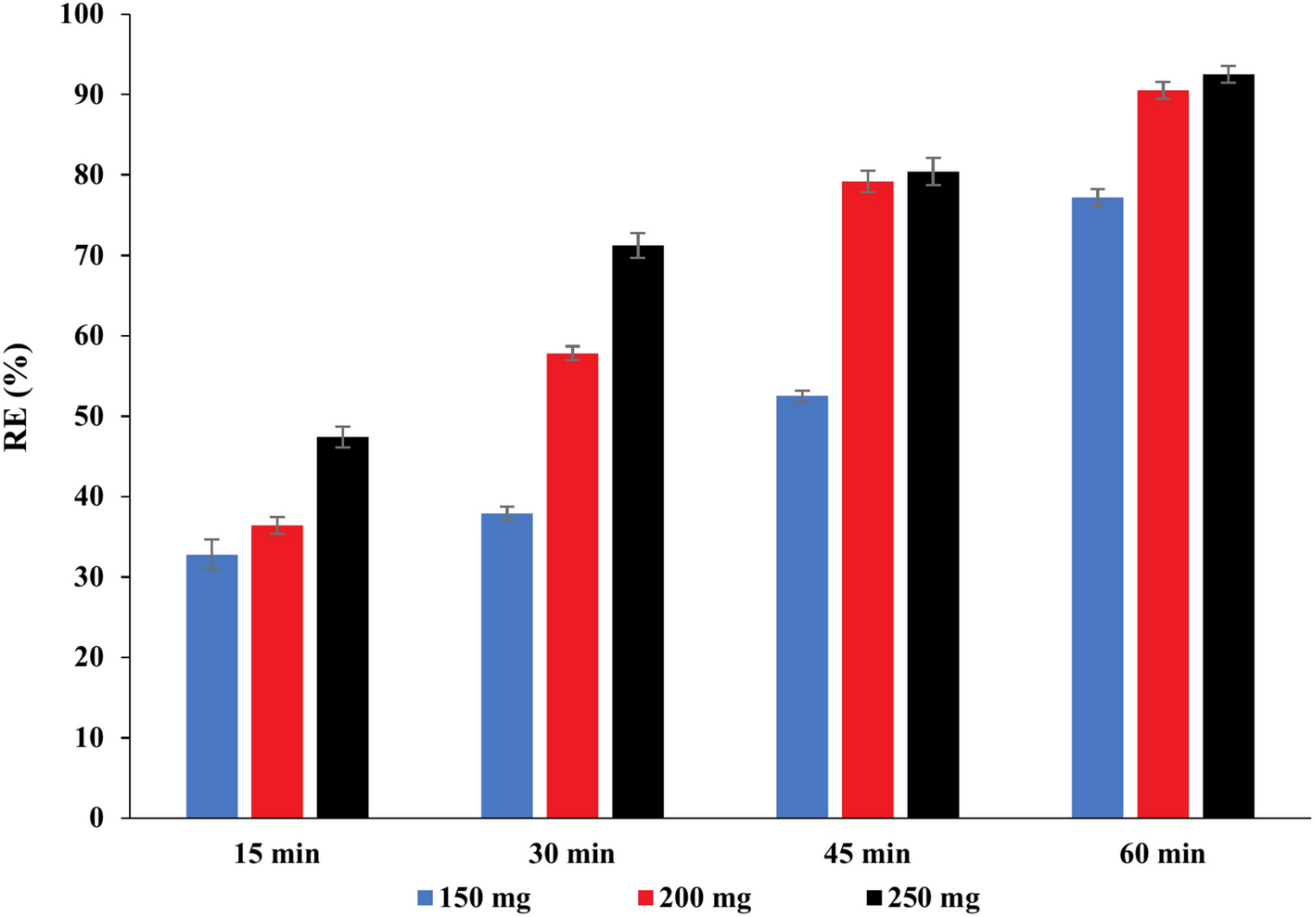
Impact of contact time and adsorbent dose on PAT removal efficiency by USOCS.

**Fig. 9:**
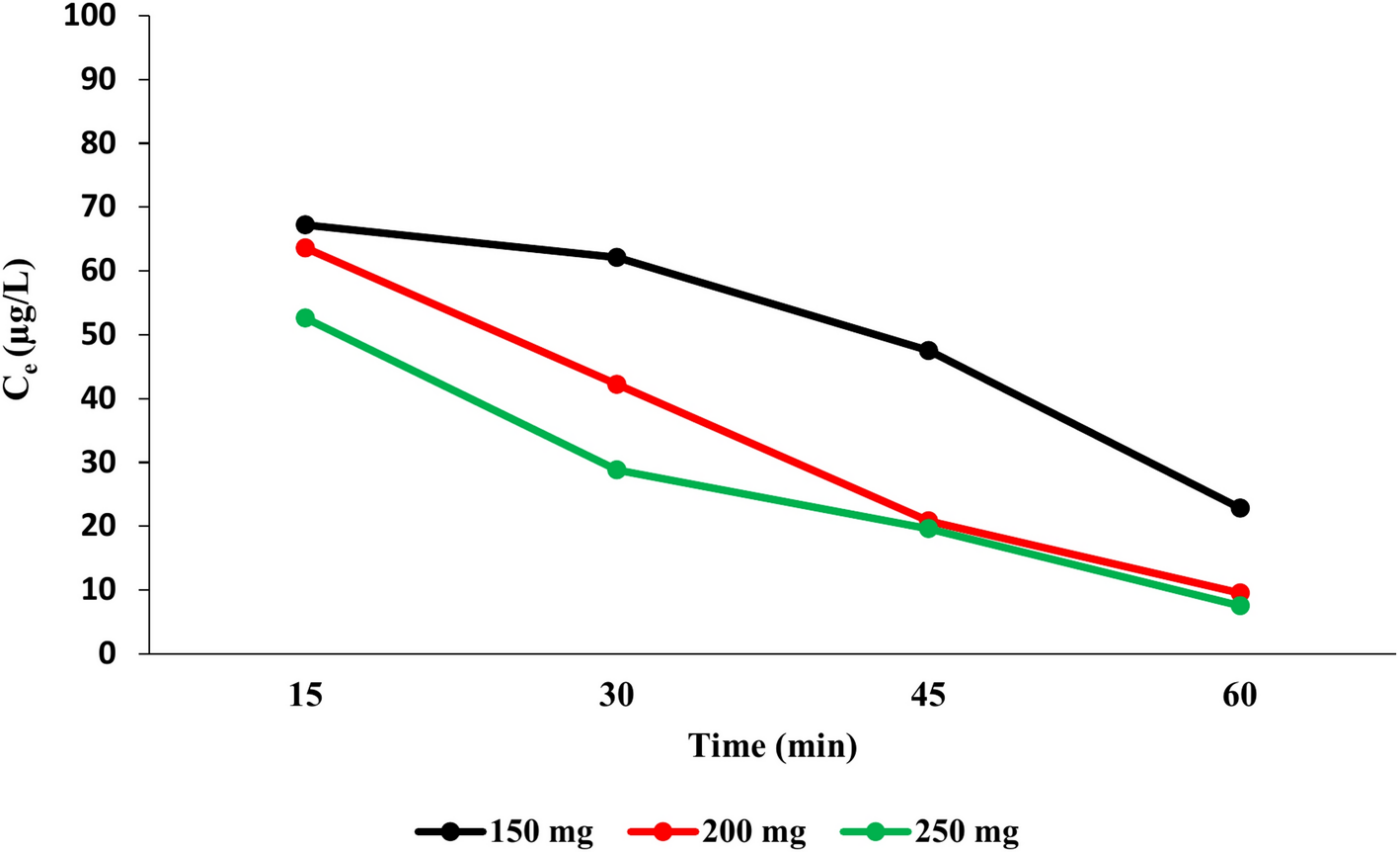
Final concentration of PAT over time after USOCS addition.

**Fig. 10:**
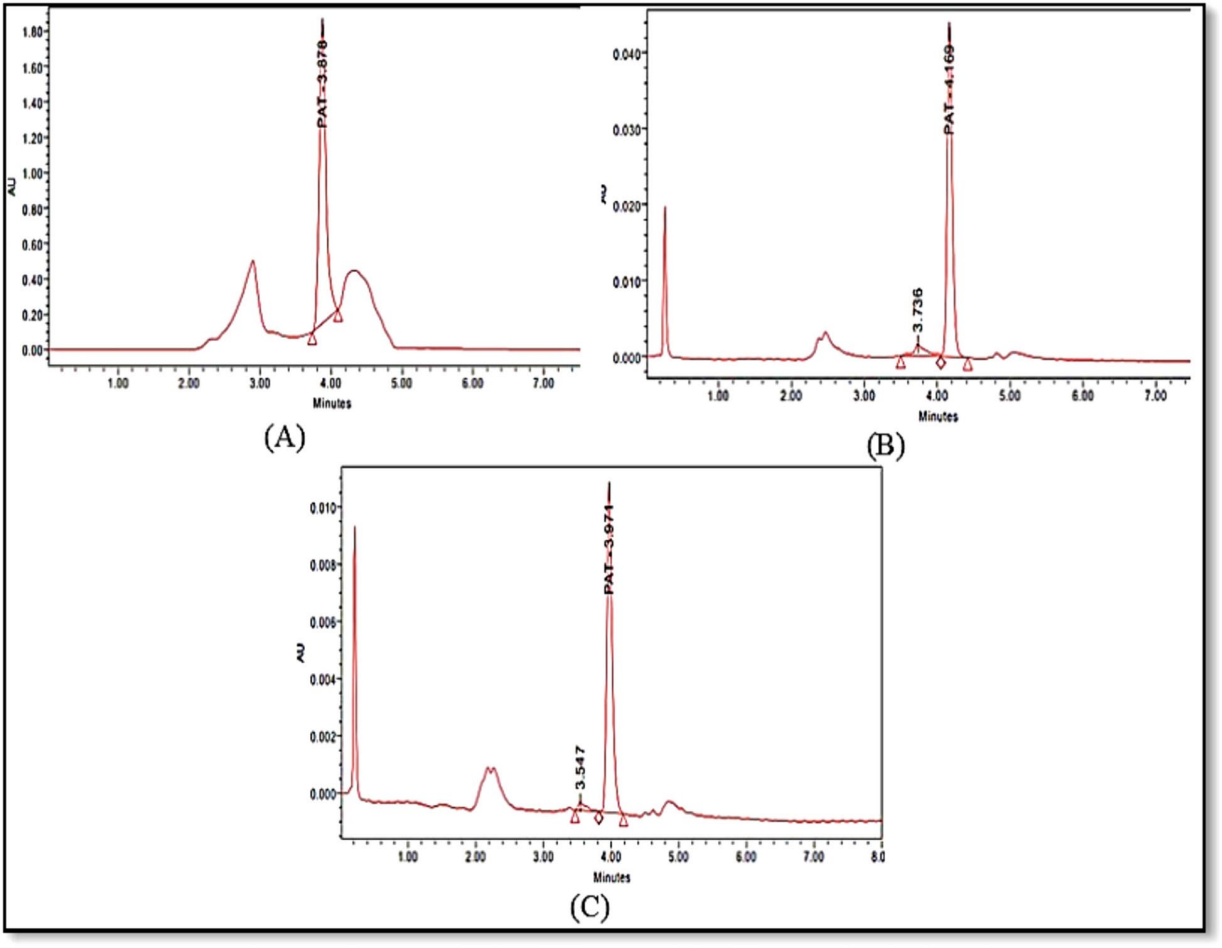
HPLC chromatogram of PAT after treatment with USOCS at 150 mg (**A**), 200 mg (**B**), and 250 mg (**C**) for 60 min.

According to the data shown in Figs. 6, 7, and 8, for all three adsorbents (NCS, OCS, USOCS), there is a clear trend showing increased removal efficiency (RE) with both longer contact times and higher adsorbent doses. The results reflected that USOCS generally yields the highest removal efficiency, indicating that modifications to the NCS by ozonation then ultrasonic may improve adsorption capacity and kinetics. RE for 150 mg, 200 mg, and 250 mg of NCS reached 29.3%, 34.20%, and 49.3% respectively at 60 min. In contrast, the OCS showed a significant increase in efficiency, especially at the highest dose, where the 250 mg dose achieved an 88.6% removal at 60 min. Similarly, the USOCS performed best, with RE of 77.2%, 90.5%, and 92.5% for 150 mg, 200 mg, and 250 mg doses respectively at 60 min. The increased surface area of USOCS allows more PAT molecules to adsorb onto the starch surface. The data display in Fig. 9 indicates a clear trend of decreasing final PAT concentrations over time for all adsorbent doses of USOCS. In addition, Fig. 10 shows HPLC chromatogram of PAT after treatment with USOCS. The presence of oxidized functional groups, such as carbonyl (C=O) and carboxyl (COOH), in the modified starch (OCS and USOCS) structure promotes stronger chemical interactions with PAT molecules. Moreover, hydrophobic interactions may also enhance adsorption efficiency by allowing the modified starch to interact with PAT. On the other hand, PAT has polar functional groups (e.g., hydroxyl and carbonyl groups) that can form hydrogen bonds with hydroxyl groups on the starch surface. Hydrogen bonding, electrostatic interactions, and hydrophobic forces are the primary drivers of adsorption, making starch-based materials effective adsorbents for PAT in aqueous solutions^45,50–52^**.** The findings of this study are consistent with previous research. Mutlu et al. (1997) found that the adsorption capacity of activated carbon for PAT increased with extended contact times^53^, while Fathi-Achachlouei et al. (2007) observed significant reductions in PAT levels with greater quantities of activated carbon powder^54^. In addition, Li et al. (2015) reported a maximum adsorption capacity of 626.4 mg/g for cross-linked chitosan beads targeting PAT at pH 7.0 and 40°C after 24 h^55^. Furthermore, Luo et al. (2017) highlighted that increasing the amount of chitosan-coated Fe₃O₄ particles led to a reduction in PAT content, with complete adsorption of PAT molecules achievable within 3 h by introducing 400 mg of magnetic chitosan to 10 mL of a 200 µg/L PAT aqueous solution^56^.

#### Kinetic study of PAT adsorption using USOCS

The adsorption of PAT onto USOCS was analyzed using both the pseudo-first-order and pseudo-second-order kinetic models to determine the adsorption mechanism. The study was conducted using different adsorbent doses of USOCS (150, 200, and 250 mg) and contact times ranging from 15 to 60 min. The temperature was fixed at 25 °C, and the PAT concentration was 100 μg/100 mL, employing the following equation. Calculating the (*q*_*t*_) of PAT at each time point can be calculated using the following formula:

$$q_{t} = \frac{{\left( {C_{o } - C_{e} } \right) \times V}}{m}$$

where *q*_*t*_ is the amount of PAT adsorbed at time t (µg/mg), C_0_ and C_e_​ are the initial and final concentration of PAT (µg/100 ml), respectively, V is volume of the solution (ml), and m is mass of the adsorbent (mg)

The linear equation of pseudo-first-order model is illustrated as follows:

$$\text{ln}{(q}_{e-}{q}_{t})=\text{ln}\left({q}_{e}\right)-{k}_{1}t$$

where *q*_*e*_ is the amount of PAT adsorbed at equilibrium, k_1_ the rate constant for pseudo-first-order kinetics( min^-1^), and t is the contact time (min).

#### Pseudo-second-order kinetic model

$$\frac{t}{{q}_{t}}=\frac{1}{{k}_{2}{qe}^{2}}+\frac{t}{{q}_{e}}$$

where *q*_*e*_ is the amount of PAT adsorbed at equilibrium (µg/mg) that take the highest *q*_*t*_ value obtained from our data at the longest time (60 min) for each adsorbent dose. *k*_2_ is the rate constant for the pseudo-second-order reaction (g/µg/min).

**Fig. 11:**
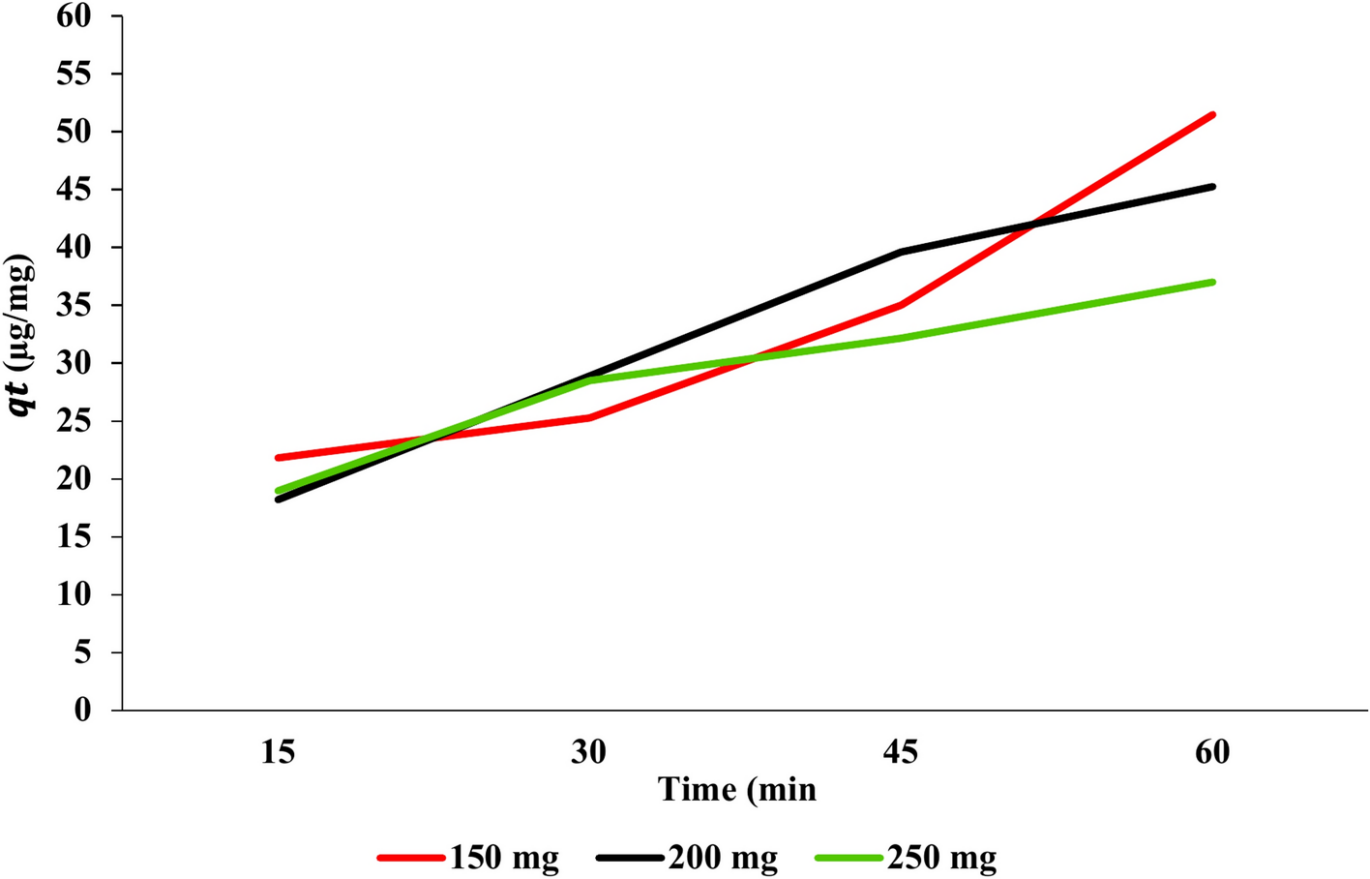
Effect of dose USOCS and contact time on adsorption capacity of PAT.

The (*q*_*t*_) of PAT onto USOCS at various adsorbent doses over time intervals (15, 30, 45, and 60 min) reveals insights into the adsorption kinetics. The values presented in (Fig. 11) show that the highest adsorption capacity (51.47 µg/mg) was recorded with a 150 mg dose at 60 min, while *q*_*t*_ at 15 and 30 min were 21.81 and 25.27 µg/mg, respectively. For the 200 mg dose of USOCS, *q*_*t*_ values started at 18.2 µg/mg at 15 min and rose to 45.25 µg/mg at 60 min. Although the initial value is lower than that of the 150 mg dose, it increased to 28.9 µg/mg at 30 min and 39.6 µg/mg at 45 min. The *q*_*t*_ values for the 250 mg dose ranged from 18.96 µg/mg at 15 min to 37 µg/mg at 60 min. Although the initial value is similar to that of the 200 mg dose, the overall maximum adsorption capacity at this dose is lower. The adsorption capacity shows a sharp increase in the initial stages before reaching a peak at 60 min, suggesting that the majority of the adsorption occurs quickly as the available active sites on the USOCS are occupied. The substantial increase in qt values suggests that the adsorbent sites become increasingly saturated over time. However, the slower increase after 45 min may indicate that the adsorption process is approaching equilibrium.

**Fig. 12:**
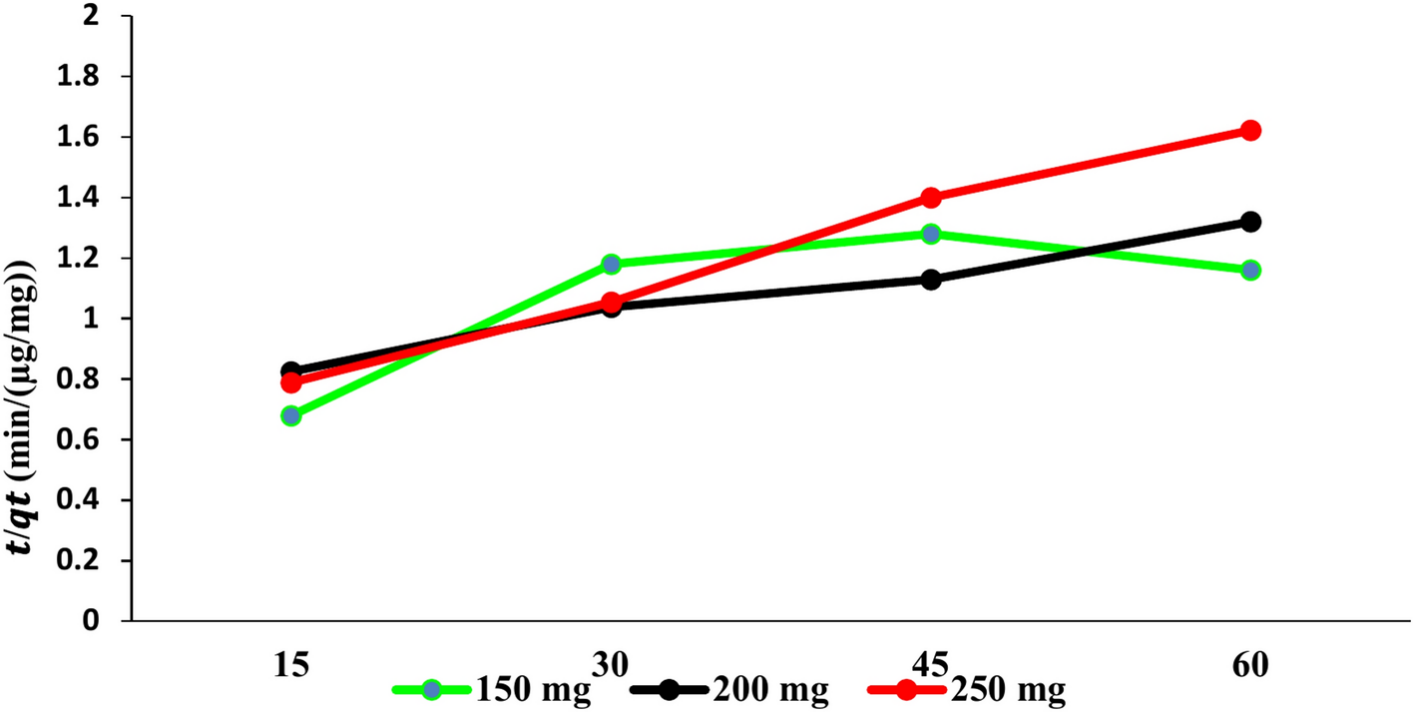
Linear relationship of (*t*) to (*q*_*t*_) in the pseudo-second-order kinetic model.

The data presented in Fig. 12 indicate that the increasing *t*/*q*_*t*_ values with time across all doses suggest a general trend. The decrease in t/*qt* with 150 mg suggests that a significant amount of PAT is adsorbed quickly, which is typical in adsorption processes. However, the fluctuation in later time points indicates that, as saturation approaches, the rate of adsorption diminishes. On the other hand, the 200 mg adsorbent dose has *t/qt* values ranging from 0.825 at 15 min to 1.32 at 60 min, demonstrating a general increase over time. The initial value is higher than that for the 150 mg dose, suggesting that the 200 mg dose has more active sites available for adsorption. The increasing t/qt values, especially at later times, indicate that while adsorption continues, the efficiency decreases compared to earlier time intervals, hinting at an approaching equilibrium. Additionally, the t/qt values for the 250 mg dose start at 0.789 and increase to 1.622. The general trend indicates that initial adsorption is rapid, with efficiency decreasing as saturation is approached. The t/qt values reflect the transition from a high rate of adsorption to a more stable state as the system nears equilibrium.

The equilibrium adsorption capacity (*q*_*e*_) decreases with an increase in adsorbent dose. At 150 mg, qe is 51.47 µg/mg, while at 250 mg; it drops to 37.0 µg/mg. This trend may be attributed to saturation effects, where an increase in adsorbent concentration leads to a reduction in the availability of PAT per unit mass of adsorbent due to competition for binding sites (Table 3).

**Table 3:** Summary of adsorption kinetics parameters for Patulin adsorption on USOCS.

Adsorbent dose (mg)	*q*_*e*_ (µg/mg)	*K*_1_ (min^−1^)	*K*_2_ (g/µg/min)
150	51.47	0.022	5.87
200	45.25	0.039	6.01
250	37.0	0.049	7.40

In addition, the results in Table 3 indicate that the increasing values of *K*_*1*_ and *K*_*2*_ with adsorbent dose highlight that both the pseudo-first-order and pseudo-second-order models can adequately describe the adsorption kinetics. However, the predominant influence of *K*_*2*_ suggests that the adsorption may involve chemisorption processes, where surface interactions become a key determinant of the rate. This implies that chemisorption is the predominant mechanism governing the adsorption of PAT onto USOCS, with stronger interactions occurring at higher adsorbent concentrations. This suggests that the adsorption rate increases, as more adsorbent is available to interact with PAT in the solution. However, the relatively low *K*_*1*_ values indicate that the process might not be entirely governed by physical adsorption. Furthermore, as the pseudo-first-order model assumes that the adsorption rate is proportional to the number of vacant sites on the adsorbent, the lower accuracy of this model suggests that patulin’s adsorption onto USOCS involves more than just surface-level interactions. The rate constant for pseudo-first-order kinetics (*K*_*1*_) increases with higher adsorbent doses. This could suggest that increasing the dose of USOCS leads to faster adsorption but reduces the overall adsorption capacity, likely due to the saturation of active sites on the adsorbent surface. The second-order model assumes that the adsorption process involves chemical bonding between the PAT and the active sites on the adsorbent surface, which aligns with the observed data. The increase in *K2* with higher adsorbent doses suggests a greater availability of active sites and stronger chemisorption interactions at higher concentrations of USOCS. This further implies that as the adsorbent dose increases, more PAT molecules can be adsorbed through stronger interactions, consistent with the theory of chemisorption dominating the process. Finally, the results indicate that the adsorption of PAT onto USOCS is likely a combination of both physisorption (weak van der Waals forces) and chemisorption (chemical bonds or complexation). The good fit of the pseudo-second-order model suggests that chemisorption plays a dominant role, possibly involving interactions between the functional groups on the modified corn starch and the PAT molecules.

### Adsorption isotherms for PAT

#### Langmuir isotherm model

**Fig. 13:**
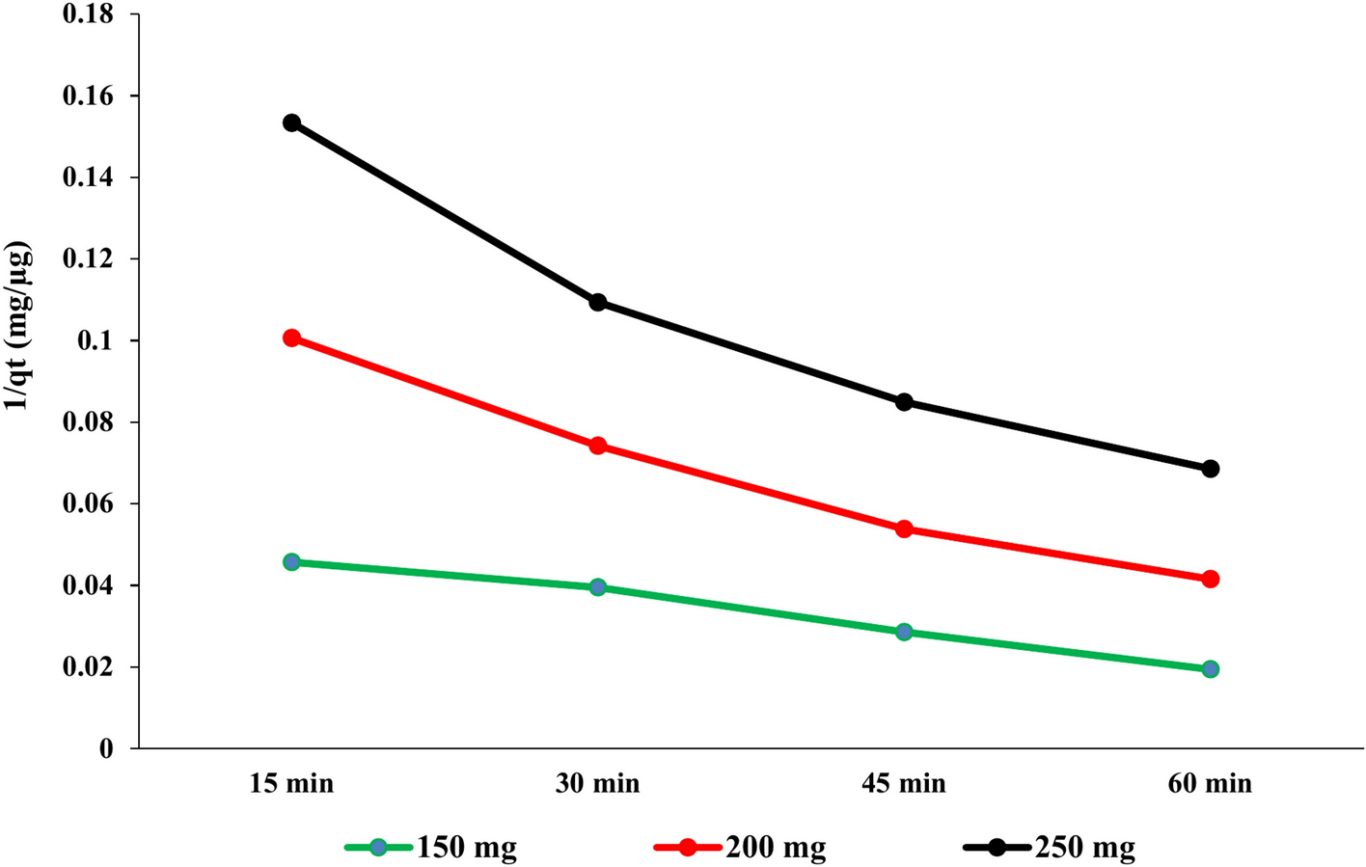
Adsorption isotherms of PAT on USOCS at different contact time.

**Fig. 14:**
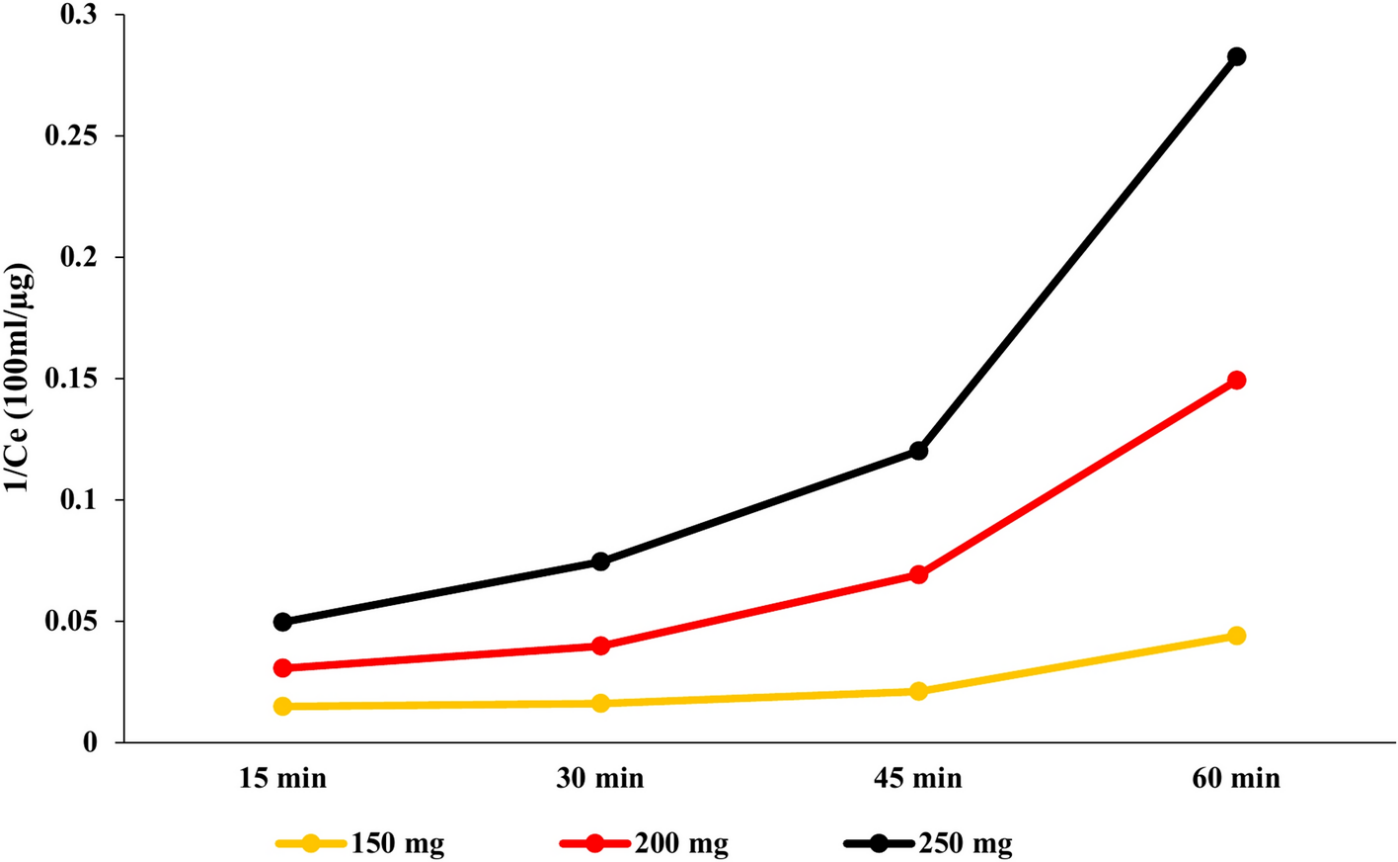
Adsorption isotherms (1/*Ce*) of PAT with Langmuir isotherm model.

The adsorption of PAT onto USOCS was studied using both the Langmuir and Freundlich isotherm models, with adsorbent doses of 150 mg, 200 mg, and 250 mg. The results of the study are summarized in Table 4 and Figs. 13 and 14, and the following conclusions can be drawn:

$$\frac{1}{{q}_{e}}=\frac{1}{ {{K}_{L } . q}_{max}}.\frac{1}{{C}_{e}}+\frac{1}{{q}_{max}}$$

where *q*_*e*_ is the amount of PAT adsorbed per unit mass of adsorbent at equilibrium (µg/mg),*Ce* is the equilibrium concentration of solute in the solution (µg/100 ml), *qmax* is the maximum adsorption capacity (µg/mg), and *K*_*L*_ is the Langmuir constant related to the affinity of the binding sites (L/µg).

**Table 4:** Adsorption isotherm parameters for PAT onto USOCS at varying adsorbent doses.

Adsorbent dose (mg)	*q*_*max*_ (µg/mg)	*K*_*L*_ (L/µg)	*K*_*F*_ (L/g)	*n*
150	1.3	19.35	1.129	1.301
200	7.16	41.92	1.113	3.579
250	15.19	54.00	1.111	6.097

#### Freundlich isotherm model

$${q}_{e}={K}_{F}.{C}_{f}^{1/n}$$

$$\text{log}{(q}_{e})=\text{log}({K}_{f})+\frac{1}{n} (\text{log}{C}_{e})$$

where *K*_*F*_ is Freundlich constant (µg/mL) and *n* = Freundlich exponent (indicates the adsorption intensity).

The results indicate a notable increase in the maximum adsorption capacity *q*_*max*_ with higher adsorbent doses. For instance, *q*_*max*_ increased from 1.3 µg/mg at 150 mg to 15.19 µg/mg at 250 mg. This trend suggests that higher doses of USOCS enhance the availability of active sites, leading to greater PAT adsorption. The Langmuir constant (K_L_) also increased from 19.35 L/µg to 54.00 L/µg, indicating a higher affinity of PAT for the binding sites on USOCS as the adsorbent dose increased. Similarly, the Freundlich constant (*K*_*F*_) remained relatively stable, reflecting consistent interaction dynamics across different doses. In contrast, *K*_*F*_ reflects the adsorption capacity of the adsorbent. The initial increase indicates that as more USOCS is added, it provides more available surface area for adsorption. However, the stabilization in *K*_*F*_ values at higher doses (200 and 250 mg) suggests that the active sites are becoming saturated, limiting additional adsorption efficiency. Higher values of *n* (greater than 1) suggest a more favorable adsorption process and indicate the heterogeneity of the adsorbent surface. As the adsorbent dose increases, the surface characteristics may change, leading to increased complexity in the adsorption sites and enhancing the adsorption mechanism. The ultrasonication process likely alters the surface morphology and porosity of the OCS, creating a larger number of active sites that facilitate the binding of PAT. This enhanced surface area directly correlates with the observed increases in *q*_*max*_ and *K*_*L*_^30,45,57,58^. The hydroxyl groups in USOCS can form hydrogen bonds with the carbonyl groups of PAT. This interaction enhances the adsorption of PAT onto the USOCS surface, as the formation of these bonds creates a more stable complex. On the other hand, Hydrogen bonds occur when a hydrogen atom covalently bonded to a highly electronegative atom (like oxygen in patulin) interacts with another electronegative atom. In the case of PAT, the hydroxyl (-OH) groups present can form hydrogen bonds with functional groups on the surface of the adsorption material. This interaction enhances the binding affinity between patulin and the adsorbent, improving the overall adsorption capacity. Also, Van der Waals Forces that arise from transient dipoles in molecules. Although they are weaker than hydrogen bonds, these forces can still significantly contribute to the overall interaction between PAT and USOCS. As the concentration of the adsorbent increases, the likelihood of van der Waals interactions also rises, further promoting the adsorption process^59–61^.

## Conclusion

This study highlights the significant potential of modified corn starch, specifically USOCS, as an effective adsorbent material for the removal of PAT in buffer solutions. The modifications involving ozonation and ultrasonic treatment not only enhanced the structural characteristics of the NCS but also introduced functional groups, such as carbonyl and carboxyl groups. These modifications resulted in an increased surface area and improved porosity, allowing for more effective interactions with PAT molecules. Additionally, it was concluded that USOCS achieved a remarkable removal efficiency (RE) of 92.5% at an adsorbent dose of 250 mg after 60 min of contact time, outperforming both native corn starch (NCS) and ozonated corn starch (OCS), which showed lower efficiencies of 49.3% and 88.6%, respectively, under similar conditions. Kinetic studies indicated that the adsorption process is primarily driven by chemisorption, with a notable increase in adsorption capacity (*qt*) over time. The maximum adsorption capacities recorded were 51.47 µg/mg, 45.25 µg/mg, and 37.0 µg/mg for adsorbent doses of 150 mg, 200 mg, and 250 mg, respectively. Finally, in isotherm analysis, *q*_*max*_ increased from 1.3 µg/mg at 150 mg to 15.19 µg/mg at 250 mg. The Langmuir constant (*K*_*L*_) also increased from 19.35 L/µg to 54.00 L/µg, indicating a stronger affinity for PAT as the adsorbent dose increased.

## Data Availability

All data supporting the findings of this research are available within the article.
